# On the Pathology, Exciting Causes, and Treatment of Bubo

**Published:** 1829-07

**Authors:** S. D. Broughton

**Affiliations:** Surgeon to the 2d Life Guards, and to the St. George's and St. James's Dispensary.


### THE LONDON
### Medical and Physical Journal.

N° 365, VOL. LXII.] JULY, 1829. [N° 37, *New Series.*

For many fortunate discoveries in medicine, and for the detection of numerous errors, the world is indebted to the rapid circulation of Monthly Journals; and there never existed any work, to which the Faculty, in Europe and America, were under deeper obligations than to the *Medical and Physical Journal of London*, now forming a long, but an invaluable series.—RUSH.

### ORIGINAL PAPERS, AND CASES,

### OBTAINED FROM PUBLIC INSTITUTIONS AND OTHER AUTHENTIC SOURCES.

### BUBO.

*On the Pathology, exciting Causes, and Treatment of Bubo.* By S. D. BROUGHTON, Surgeon to the 2d Life Guards, and to the St. George's and St. James's Dispensary.

*General Observations.*

THERE is no species of tumor so frequently presented to the surgeon's notice, and at the same time so troublesome to manage, as the common bubo, or enlarged glands in the groin and axilla. In military practice, (especially among cavalry soldiers,) and with the labouring classes, which form a considerable portion of dispensary patients, this complaint is most often encountered. Its course is usually marked by an uncertain and variable character, and is productive of more or less constitutional irritation, while it entails a long and tedious suspension of daily duties and labours. Interposed between the trunk and the limbs, every movement of the latter irritates and inflames the tumor and the surrounding integuments. Emaciation and debility generally ensue from this state of the parts affected, and not unfrequently fever attends, so as to render repose, with cooling and soothing remedies, absolutely necessary. The absolute absorption of morbid matter, and its conveyance into the body of the gland, its positive existence there as an immediate exciting cause, and the alleged infectious nature of the suppuration in glands so excited, appear to be points resting entirely upon hypothetical bases. It may, indeed, be very fairly questioned whether such considerations should influence our treatment of buboes, upon the ground that, whatever might have been the remote cause, their appearance is unconnected with it, but as a secondary effect or consequence. The ordinary result of the dispersion of a bubo, even after suppuration has taken place, seems to warrant and support this question.

In the greater number of cases commonly met with, the bubo probably arises from some local source of irritation, mechanical or otherwise, and remote from the apparent glandular affection. Thus, an irritable corn, or pressure from a tight boot or shoe, a scratch or excoriation of the skin, or any other such simple cause, acting upon parts in the absorbent line of direction leading to glands, is just as likely to excite the enlargement of one or more of them, as the introduction or formation of virulent matter; nor are the signs which buboes exhibit from either source in any way peculiarly characteristic of the original exciting cause, although their subsequent appearance may perhaps become modified by constitutional diathesis; as, for example, in a patient of a naturally strumous habit. In whatever manner the bubo may be produced, its occasional progress indicates the necessity of an early attention to the means best calculated to disperse it in the incipient stage, in order to obviate suppuration, as well as to prevent that indolent and indurated state often assumed, and in which its prolonged continuance is a source of much annoyance and discomfort to the patient.

*Of the Parts immediately involved in the Formation of Bubo.*

If the formation of buboes be carefully watched, we shall find that the glands themselves are the first and immediate seat of inflammatory action, and this condition is not commonly traceable along the course of the lymphatic vessels connected with the affected glands: frequently, indeed, the remote exciting cause is looked for in vain. Usually the mere enlargement of a single gland is not productive of much pain and irritation, until the surrounding cellular connexions become involved, and the throbbing of the neighbouring cutaneous vessels renders the integuments tense and tumefied. The little degree of nervous development in these glands may account for this absence of pain at first, and their relations to other organs by means of nervous filaments point out the source of the general sympathy which they hold in common with the system, while the sensibility and profusion of the nerves of the cutis account for the increase of pain and tenderness following the progress of the inflammatory stage. Although sometimes the cellular inflammation extends far around, and an erysipelatous action occasionally supervenes, yet the inflammatory condition of the parts is most commonly circumscribed.

*Proximate Cause of Bubo.*

The proximate cause of this condition of the parts affected appears to be obstruction to the free course of the lymph. Whether on this occasion the fluid may hold suspended in it any virulent substance or not, seems to be a question relating to a very doubtful fact. The probability appears to me to be, that the *proximate cause is irritation only*, and *not absorption of virus ;* and *that the latter, when existing, is only the remote cause.* The lymphatic obstruction probably commences in the capillary vessels, producing distention of the veins and arteries; or a state of *atony* may be said to belong to the vasa efferentia of the conglobate glands, induced by a distant source of irritation ; and the lymph thus obstructed is poured into the surrounding cellular substance, the finer particles escaping by transpiration, and the grosser adhesive matter remaining to amalgamate the cells with the adjacent texture, so as to limit the overflowing of the lymph and to circumscribe the inflammatory action, while the hardness and tumescence in the parts themselves are thus increased. In this condition the glands so placed are most likely irrecoverably lost, and their functions transferred to others; the fluid lymph becomes dispersed, and the tumor dwindles to a small, permanently hard, and round ball.

*Of the Suppuration of Buboes.*

When the inflammatory action is incontrollable, suppuration occurs in the centre of the tumor, and the surrounding walls of matted and condensed cellular texture perfectly enclose the pus, and thus the abscess is contained in a sac. This process is gone through with different degrees of activity and celerity: when slowly conducted, the pus may often become dispersed, and the tumor so reduced to the natural level of the integuments around. But, when the formation of pus is rapid and copious, the irritation from overstrained skin is excessive, and relieved only by a spontaneous discharge of its contents, or by an artificial opening.

Excepting the pain and irritation from pressure, and the straining of sensible parts, the presence of the pus, or even its reäbsorption, appears to be innoxious to the system; unless, indeed, the stagnant collection, by exposure to the atmospheric air in an open bubo, should generate carburetted and sulphuretted hydrogen gas, when the altered qualities of the pus become the exciting cause of serious disorder in the constitution.

*Of the Progress and Termination of Buboes generally, and their exciting Causes.*

To form any certain prognosis of the termination of a bubo in its early stage seems to be impossible, so variable is its general character and appearance. When active and irritable, it will suddenly become passive and stationary; or, when to outward view indolent, it will occasionally suppurate quickly, without stretching the integuments, and thus creating no pain or irritation.

I am unaware of any specifically marked signs by which the terminations are to be prognosticated, until the eve of their occurrence, either local or constitutional. The agency by which the changes are affected lie concealed, and the vicissitudes which buboes go through have, in consequence, a capricious appearance. These changes do not seem to depend directly on the gland itself, for, throughout the different stages of buboes, the glands remain much in the same state and size. The changes seem to be rather referrible to the integuments and cellular substance over the inflamed gland, and these run into inflammation more or less extensively, assuming different shades of redness, sometimes having a rosy, sometimes a bluish, and at others a brownish tint, probably from constitutional peculiarities rather than any which may be connected with the ultimate exciting cause.

From whatever source a bubo may originally proceed, we seem to have no proof that its progress and termination are influenced by it. A mild gonorrhœa of short duration, accumulation of viscid mucus behind the glans, a phymosis, an injury of the foot or toe, &c. are followed by buboes, similar in their appearance, continuance, and termination, to those arising from the most virulent gonorrhœa, or the inoculation of poisonous matter. I should imagine the buboes of the plague itself to be influenced by the state of the constitution, and not by the virus supposed to be imbibed. When, therefore, we hear of the malignant, the mild, the sympathetic, the syphilitic, or the strumous bubo, I should be disposed to refer all these characteristic distinctions equally to constitutional influence, to the disorders of health, and not to the nature and properties of the ultimate exciting cause; and, as such peculiarities are not usually pre-evident, the prognosis of bubo is seldom certain.

*Of the Treatment of Buboes generally.*

The foregoing views of the pathology and exciting causes of bubo have been introduced with the view of adapting the treatment of this tumor to the principle suggested. Accordingly, the first indication seems to be to remove the irritation derived from the presence of an enlarged and indurated gland in the groin or axilla; the means most suited to which end appear to be such as at once diminish and sooth the swollen parts, and these are of a topical kind. If pain and fever exist, local applications will thus tranquillize the system, in conjunction with repose in a recumbent position. The choice of local remedies must be governed by the state of the parts. The active and irritable state requires one species of application, and the indolent, sluggish, and insensible state, another. The secondary state of bubo, that of established suppuration, may be considered in the same light, and requires its treatment to be adapted to its immediate condition. Great attention seems to be necessary to the secondary state, as upon the choice of remedies depends much of its subsequent condition and duration.

*Treatment of Bubo in its primary State.*

If the incipient bubo be active and irritable, the remedies should be directed to the tranquillizing of the parts, and the adoption of such measures as may best secure the end in view. The inflammation of cellular surfaces seems to be beneficially treated with cooling, astringent, and evaporating lotions; but, in glandular inflammation, such remedies appear to be insufficient, if not injurious. They are not calculated to relax and soothe the indurated and irritable gland, to check its swelling and suppuration, or diminish pain, although they lessen the inflammatory action of the softer and more yielding parts around, and contract the limits of inflammation within the sphere of the tumor, which advances in about the same ratio. The application, also, of leeches in the incipient state appears to aggravate the condition of the tumor, so that the advantages of abstracting blood seem to be counterpoised by the irritation of the leechbites. Expetience has led me to discard the use of cold lotions and leeches, as inadequate to secure the fulfilment of the objects in view, and rather tending to increase than relieve the existing symptoms, and promote the advancement of the tumor, instead of its repulsion, ultimately. A moderate and equable degree of warmth, combined with moisture, is more calculated to soothe the parts, repress their activity, relax the induration, and finally disperse the tumor. To this end, fomentations thrice a day, succeeded by light, warm, and moist poultices of linseed meal, or finely grated bread, or both mixed together, and soaked in boiled milk and water, may be advantageously applied; and, if these be insufficient to allay pain, the fomentations and poultices may be made of the decoction of poppyheads and conium. The first appearance of enlarged glands will often yield merely to fomentations, if the body be kept in repose. The result of such a principle of treatment, when the tumor subsides, is not attended generally, as in cases after the use of cold applications, with a kind of marble-like ball left in the groin or axilla, and liable to subsequent relapse. The dispersion in one case is more complete than in the other. Where the tumor is not active and irritable, its advance may sometimes be checked by cold washes; but it is then liable to become stationary and indurated; an effect not resulting from the use of fomentations and poultices.

With regard to constitutional remedies, such as tend to diminish the action of the heart and arteries, to cool the system, and assist the suppression and dispersion of the tumor, are indicated; and, in doses proportioned to the heat of the skin and fever, the invaluable remedy of tartarised antimony, with saline mixture and Epsom salts and senna, I have always found to be highly useful adjuncts, and far preferable to the use of narcotics, where pain attends the incipient advance of bubo, excepting as local applications. And, where plethora is present, bleeding from the arm will always tend to check the inflammatory stage, with general advantage to the system. But, when fever does not exist, and the tumor is not actively inflamed, I see no advantage in the use of antimony, &c.: on the contrary, in sluggish cases, the reducing plan may perhaps be injurious rather than beneficial.

It occasionally happens that the incipient stages of bubo are not attended with tension of the superincumbent integuments, which lay more loosely over the surface of the tumor. But this state of the parts may soon become altered, if repose be not enjoined, and the case thus become prolonged and more complicated. This passive state is also more aggravated by cold applications and leeches; but it is less likely to be completely reduced by mild, moist, and warm applications, than the more irritable and active tumor. When these, therefore, have apparently brought the tumefied parts into a perfect state of passiveness, and the induration and swelling yield no further, blisters repeatedly applied, or cautious and gentle friction with the Linimentum olei camphorati, to which the Unguentum hydr. may be usefully added, often tend to the gradual removal of this chronic state. Should too much action be excited, the fomentations and poulticing may be resorted to again beneficially. If the first intention of stimulating remedies be not fulfilled, they have this advantage, that they bring the tumor forward to suppuration, and thus afford the means of destruction, if we cannot disperse it, and so prevent the inconvenience and liability to relapse which belong to indurated and enlarged glandular tumors situated in the groin or axilla. With labourers and soldiers, I have in such cases generally recommended a return to labour and duty until active inflammation be excited, and a chance thus afforded of the permanent removal of a troublesome and inconvenient affection.

*Treatment of Bubo in its secondary State.*

When the measures which I have recommended to be pursued in the primary state of bubo do not succeed in preventing its advance to the secondary state, and suppuration is inevitable, whether cold or warm applications, mild or stimulating, are to be preferred, cannot then become a question. The same treatment which is calculated to disperse the tumor will now facilitate and concentrate the process of suppuration, diminish pain, and often tend to produce a speedy, favorable, and spontaneous discharge of the matter formed. Perhaps, when such is the termination indicated, it is best not to interpose with an artificial opening, and, with careful and judicious attention and management, the abscess will not long continue to be troublesome afterwards, where the constitution is in good order. The accumulation of stagnant matter should be avoided while the original aperture remains open, and a mild emollient poultice applied thrice a day, accompanied with gentle and gradually increased pressure, is well calculated to promote the discharge of the pus, prevent the formation of sinuses, and encourage the adhesion of the parietes of the sac; and, when this cannot be brought about, but a weeping orifice continues, the sac may be irritated, or even destroyed, by stimulants or escharotics.

In this stage of the secondary state, a generous diet, regular mode of life, and good air, are as essential to the patients as are a low diet and reducing measures in the primary state. But, if the progress of suppuration has not been accompanied by high inflammatory action of the parts involved, spontaneous discharge is either procrastinated or altogether avoided, and a soft fluctuating feel is presented to the touch, on applying the fingers to the surface of the bubo. We have then an opportunity of fairly employing surgical skill; and, in the choice of several means in our power, will probably depend the permanence and celerity of the cure. In some cases the quantity of pus becomes gradually diminished while poultices are being applied, but rarely entirely removed, although I have seen this effected. In such cases, where a languid state of the parts around exists, I have known the dispersion of the pus accelerated by repeated blistering. The same result may also be sometimes produced by the cautious and gradually increased pressure of a flannel bandage, passed across the tumor, and round the loins and thigh. I have also used, in similar cases, poultices mingled with mercurial ointment and soft soap, with benefit. But if there seems to be no indications of such measures effecting the removal of the pus, (and if the skin over the tumor be very thin, and the accumulation below large, these will not succeed, and the integuments will let out their contents,) it appears preferable to make an artificial opening in one of these modes, either by a simple puncture, a long incision, or the application of caustic so as to form a deep and wide eschar. Of these methods experience has taught me, certainly in general, to reject the first; for, though sometimes speedily successful, it most frequently entails a long continuance of the abscess in a state of secretion and discharge, requiring some trouble to manage. The two latter operations appear to be greatly preferable; and of these, perhaps, the formation of an eschar by caustic is the most effectual of the two, and the readiest mode of ensuring an entire and permanent destruction of the bubo, usually not long after the casting off of the slough. The manner in which I prefer applying the caustic is to rub the skin covering the bubo with the Kali purum dipped in a drop of water, till the parts to the extent of about a shilling are sufficiently affected to ensure the destruction of their vitality. An emollient poultice, mingled with narcotic substances, will diminish the temporary pain from the action of the caustic, and facilitate the removal of the eschar formed, and part of the pus will flow out and part be conveyed away within.

*Treatment of the third state of Bubo.*

Unless the constitution be affected with any morbid diathesis, the parts exposed generally granulate and cicatrize speedily with the application of cold, astringent, or stimulating solutions, simple or digestive ointments, according to the appearances of the sore, in conjunction with pressure by a proper bandage, or of mild poultices should inflammation occur; while the carrot poultice may be useful if the ulcer take on a sloughing character.

When the parts treated with caustic recover from the first state of irritation into which they are thrown, the patient taking moderate exercise promotes, rather than impedes, the healing. An irritable state of the sore sometimes supervenes, and this seems greatly relieved by sedative and caustic solutions.

I have already considered the relation of the second, or suppurative, stage of bubo to the remote cause in a pathological point of view, and in my remarks on its treatment I have not been at all guided by any consideration of the necessity of regarding it as influenced by a specific virus. The leading principle of treatment required appears to be the same in all cases; the local means only by which the general objects may be secured varying according to existing circumstances.

I consider the distinctions between what are termed scrofulous and syphilitic buboes to lead to no practical result as to local treatment, nor indeed as to constitutional remedies, any further than, whatever may be the diathesis of the patient, and the constitutional state of irritation, debility or otherwise, we cannot reasonably expect the third state of bubo, or that in which it is opened, either naturally or artificially, to heal so readily without meeting the constitutional indications. These require it to be met, perhaps, by regulating the diet, improving the air in which the patient breathes, correcting the state of the digestive organs, and promoting health and strength by general means. When bark and acid agree with the stomach, and the decoction of sarsaparilla and extract do not nauseate or oppress the patient, very beneficial results appear to follow their use. Mercury, in every stage and state of bubo, appears to me to be generally inadmissible or useless, excepting in the topical manner recommended, and indeed it is often injurious. Some obstinate sores may be improved in condition by the application of mercurial washes and ointment, and such I have often observed to be the case with those which follow the opening of buboes. The third state of bubo I would treat as I would treat any other case of ulceration rendered difficult to heal from scrofulous or any other diathesis; nor do I see any necessity or advantage likely to arise from considering the open buboes resulting from venereal complaints in any other light, or treating them upon any other principle than that of meeting their general indications, without reference to their remote cause.

It very frequently happens that the healing of open buboes is retarded by the presence of an enlarged gland in the centre of the sore, or towards one end. No remedies, constitutional or local, will then succeed in inducing entire and permanent cicatrization. The only remedy in this case which I have found to be effective is the destruction of the gland. The loss never entails the least inconvenience whatever; and the safest and most complete method of destroying it is by the periodical introduction of small pyramidal troches into its substance, composed of minium, amalgamated into solid masses about an inch long with solution of gum arabic. Their number should be gradually increased, and in a short time the gland will be thus removed from any further obstruction to the healing of the sore, which then readily takes place.

*Concluding Remarks.*

From the result of some years' observation and experience, I am led to conclude that the treatment of buboes needs not to be influenced by a consideration of the remote cause, until the sores established in their open state become affected by some constitutional diathesis, or morbid condition of irritability or otherwise, when the ordinary measures recommended for the alteration of the diathesis, or restoration of the system to health, will afford the most rational source from which the healing of the sore may be finally expected.

The mild or malignant character of a bubo I consider to be dependent on constitutional peculiarities and accidental circumstances; and, while its various phenomena require different methods and modifications of treatment, no advantage can be expected to result from any consideration of the remote exciting cause, any further than its removal as a continued source of irritation while it remains. I would encourage the mild bubo with gentle means, irritate or stimulate the indolent bubo, and soothe and tranquillize the bubo of the malignant kind, and in all cases regard the constitutional diathesis as a frequent immediate cause of any difficulty which may exist in the final destruction or healing of the bubo, excepting where any such obvious obstruction as that of a diseased gland presented itself to my notice.

12, *Great Marlborough street; May* 15, 1829.

---

TYPHUS AND VARIOLA.

